# Rapid Nanopore Assay for Carbapenem-Resistant *Klebsiella pneumoniae*

**DOI:** 10.3389/fmicb.2019.01672

**Published:** 2019-07-30

**Authors:** Haofu Niu, Weili Zhang, Liangwan Wei, Meng Liu, Hao Liu, Changjian Zhao, Peng Zhang, Quanfeng Liao, Ya Liu, Qingyue Yuan, Siying Wu, Mei Kang, Jia Geng

**Affiliations:** ^1^Department of Laboratory Medicine, State Key Laboratory of Biotherapy and Cancer Center, West China Hospital, Sichuan University and Collaborative Innovation Center, Chengdu, China; ^2^Department of Microbiology, West China School of Basic Medical Sciences and Forensic Medicine, Sichuan University, Chengdu, China

**Keywords:** nanopore assay, carbapenem-resistant *Klebsiella pneumoniae*, rapid clinical detection, label-free, low-cost

## Abstract

The prevalence of carbapenem-resistant *Klebsiella pneumoniae* (CRKP) is rapidly increasing worldwide in recent decades and poses a challenge for today’s clinical practice. Rapid detection of CRKP can avoid inappropriate antimicrobial therapy and save lives. Traditional detection methods for CRKP are extremely time-consuming; PCR and other sequencing methods are too expensive and technologically demanding, making it hard to meet the clinical demands. Nanopore assay has been used for screening biomarkers of diseases recently because of its high sensitivity, real-time detection, and low cost. In this study, we distinguished CRKP from carbapenem-sensitive *K. pneumoniae* (CSKP) by the detection of increasing amount of extracted 16S ribosomal RNA (16S rRNA) from bacterial culture with antibiotics imipenem, indicating the uninhibited growth of CRKP by the imipenem. Specific signals from single channel recording of 16S rRNA bound with probes by MspA nanopore allowed the ultra-sensitive and fast quantitative detection of 16S rRNA. We proved that only 4 h of CRKP culture time was needed for nanopore assay to distinguish the CRKP and CSKP. The time-cost of the assay is only about 5% of disk diffusion method while reaching the similar accuracy. This new method has the potential application in the fast screening of drug resistance in clinical microorganism samples.

## Introduction

*Klebsiella pneumoniae* (KP) is one of the most important opportunistic pathogens in the clinical infection (Debby et al., 2012; Aracil-Garcia et al., 2017). KP usually presents in the intestines of humans and animals (Saidel-Odes et al., 2012). The infection of KP can cause serious clinical consequences, including central nervous system infection or abdominal infection, etc. (Khan et al., 2014; Chew et al., 2017). Antimicrobial agents are the major treatments for KP infection; early and proper using of antimicrobial agents is the key to KP infection cure (Nepka et al., 2016; Su et al., 2018). However, the widely use of broad-spectrum antimicrobial leads to the strong antimicrobial resistant characteristics of KP (Rojas et al., 2016; Lomonaco et al., 2018), which results in the prolonged course and failure in treatment. The carbapenem-resistance of *K. pneumoniae* HS11286 (PMID: 26169555) (Bi et al., 2015) may be caused by biofilm formation, active antimicrobial efflux, and β-lactamase generation (Chew et al., 2017).

Accurate and fast diagnosis of antimicrobial resistance of KP in the infected patients is very important for treatment, as it can help doctor choose proper kinds of antimicrobial agents, reduce the treatment cycle, and improve prognosis. Bacterial drug resistance phenotype detection, β-lactamase detection (Rojas et al., 2016), and drug resistance gene detection (Chiu et al., 2017) are the current major methods for drug resistance detection. The detection of bacterial drug resistance phenotype need sufficient time of KP culture, which is usually time-consuming; β-lactamase detection is fast, but it has a relative small detection range (Whichard et al., 2010; Rood and Li, 2017); gene detection for drug resistance has high precision, but it is costly and time-consuming as well (Frickmann et al., 2014).

**Figure 1:**
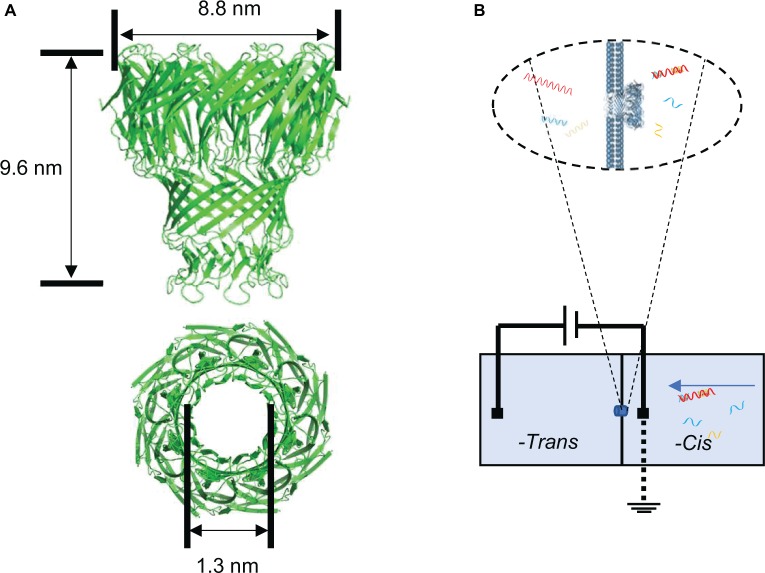
Structure of the nanopore used in the study and single-channel recording setup for nanopore assay. **(A)** Side view of MspA nanopore (top) and top view of MspA nanopore (bottom). **(B)** Schematic diagram of the single channel recording setup for 16S rRNA-probe complex detection.

Nanopore sensing is a novel biosensing and biodetection technology with single molecular sensitivity (Kasianowicz et al., 1996; Branton et al., 2008; Wanunu, 2012), which contributes to its widespread use in the third-generation DNA sequencing. The nanometer-sized protein pores are embedded in a phospholipid membrane, which separates the chambers into two parts (*cis* and *trans*). When a voltage is applied across the chamber containing a certain concentration of ion solution, the charged detection substance in the system is driven through the pores by the voltage to the other chamber. The patch clamp sensor detects the current change signals of the nanopore (Manara et al., 2015). Different molecules transported through the nanopore can cause corresponding blockage signals of the current flow (Howorka and Siwy, 2009; Ying et al., 2013). Using the specific translocation signal and translocation frequency, qualitative and quantitative analysis of the detected molecules can be achieved (Kim and Gale, 2008; Yang and Yamamoto, 2016). This nanopore sensing technology has the advantage of label-free, fast, real time, and high sensitivity with small volume of samples needed (Wang et al., 2013) in the detection process. These features are suitable for the rapid diagnosis of disease and detection of biomarker (Wang et al., 2011). The protein mycobacterium smegmatis porin A (MspA) nanopore is one of the outer membrane proteins of mycobacteria with a length of 9.6 nm and a diameter of 1.3 nm (Figure 1A; Faller, 2004). The nanopore is highly efficient to be incorporated into bilayer lipid membrane (Figure 1B) and allows the translocation of single-strand nucleic acids through the pore (Butler et al., 2008; Fleming et al., 2017). Due to its short and narrow channel constriction, MspA nanopore is ideally suitable for nanopore sequencing (Laszlo et al., 2016) such as identifying single nucleotides within random DNA (Manrao et al., 2011) or detecting and mapping 5-methylcytosine and 5-hydroxymethylcytosine within single strands of DNA (Laszlo et al., 2013).

**SCHEME 1:**
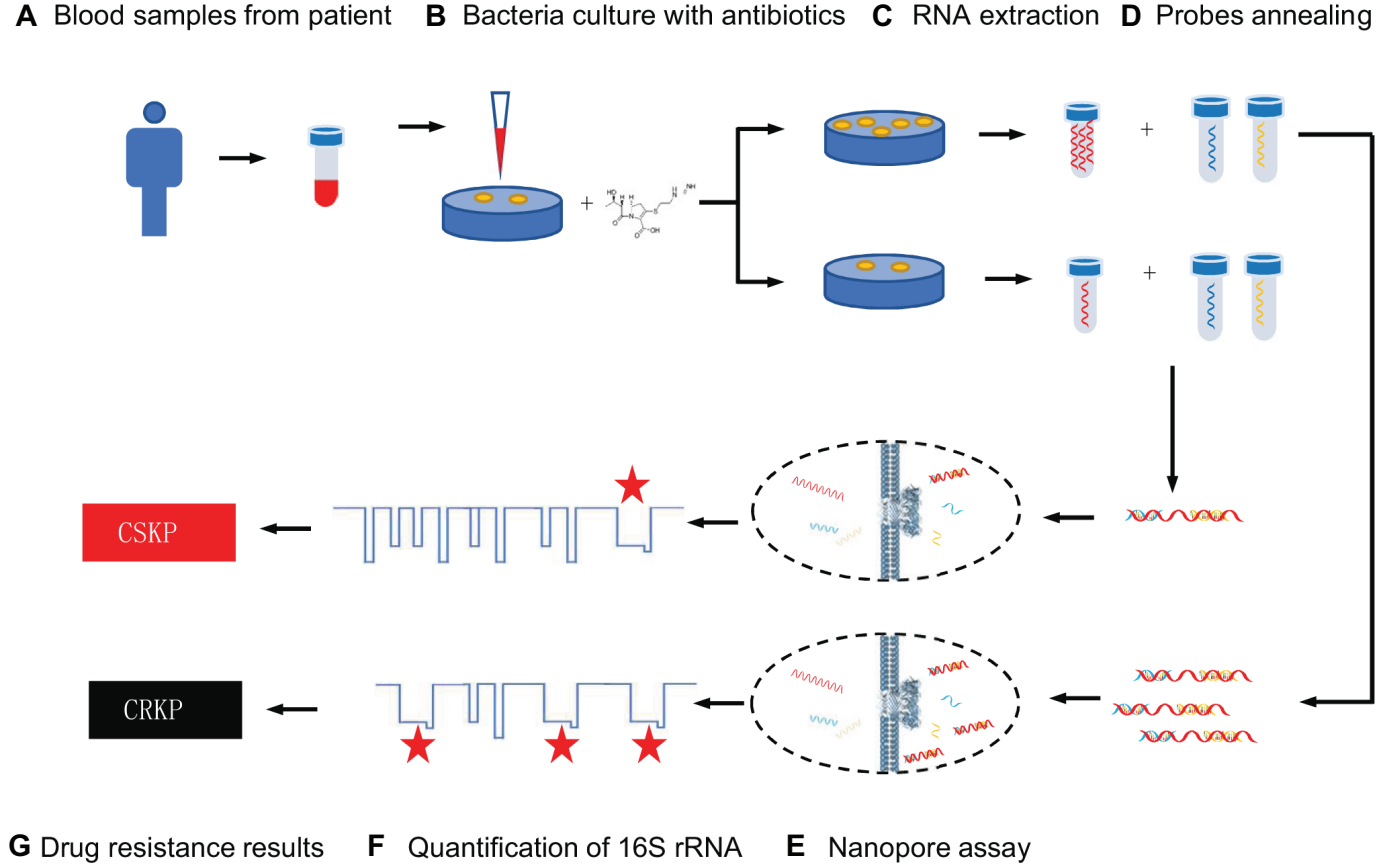
Procedure of nanopore assay for carbapenem-resistant *Klebsiella pneumoniae*.

16S rRNA that presents in all bacteria is a component of the 30S subunit in the ribosome of prokaryotes, and its function does not change over time (Patel, 2001). 16S rRNA can be used to identify bacterial species because it contains a highly conserved region shared by all bacteria and a hypervariable region with differences in different bacteria (Kolbert and Persing, 1999; Pereira et al., 2010; Srinivasan et al., 2012). It has been proven to be a reliable genetic marker and is often used for bacterial classification (Liu et al., 2012; Deurenberg et al., 2017). There is literature demonstrating that it can be used for the identification of clinical pathogens (Schuurman et al., 2004; Srinivasan et al., 2012). In this study, we provide a novel, efficient, and rapid method based on the MspA nanopore to distinguish the CRKP and CSKP at the single molecular level (Scheme 1). These strains which identified by MALDI-TOF MS are separately cultured with imipenem for several hours. 16S rRNA is highly conserved and specific, which has become a powerful tool for pathogen detection and identification in the genetic testing technology (Kolbert and Persing, 1999; Liu et al., 2012; Deurenberg et al., 2017), so it was chosen as the parameter for the measurement of amount of live KP after culturing under the antibiotics. We use specific probes to combine with 16S rRNA in *K. pneumoniae* and record the nucleic acid reading processes by nanopore assay. The frequency of specific signals regarding the translocation of the target nucleic acid through nanopore reflects the quantities of live KP. Therefore, a quantitative analysis of remaining live KP against carbapenem can be established. We prove that only 4 h of bacterial culture was needed for the discrimination when using 4 MCF as the initial concentration of bacteria suspension. The accuracy of this method is 90%, and the time-cost is far less than cell culture method.

## Materials and Methods

### Materials

4-(2-Hydroxyethyl)piperazine-1-ethanesulfonic acid (HEPES, purity > 99.5%, CAS#7365-45-9), potassium chloride (KCl, purity > 99.0%, CAS#7447-40-7), agarose (purity > 99.0%, CAS#: 9012-36-6), chloroform (purity > 99.0%, CAS: 67-66-3), isopropyl alcohol (purity > 99.0%, CAS#: 67-63-0), and alcohol (purity > 99.0%, CAS#: 64-17-5) was purchased from Sigma-Aldrich. RNase inhibitor (5 KU), pET-28b plasmid, and all the DNA were provided by Sangon Biotech. 1,2-Diphytanoyl-sn-glycero-3-phosphocholine (DPHPC) was purchased from Avanti. PrimeSTAR HS DNA polymerase was purchased from TaKaRa. Imipenem (CAS#: 64221-86-9) was purchased from MSD.

### Preparation of Bacterial Extracts

Two groups of *K. pneumoniae* samples from clinical patients were provided by West China Hospital of Sichuan University. The KP samples were cultured to two different concentrations. The concentration of first group was 0.5 MCF, and the second group was 4 MCF. The final concentration of imipenem used in two groups was 16 mg/L at the beginning of culture. The total RNA of KP was extracted by TRIZOL method. First, 100 μl of bacteria solution is collected. The supernatant was removed after centrifugation subsequently (8,000*g*, 4°C, 2 min). The precipitation with lysozyme was incubated for 10 min at 37°C. The KP cells were lysed, and the total RNA was extracted and washed with ethanol. The centrifugal tube cap was removed, and the tube was dry at room temperature for 5–10 min, then the DEPC water was added or dissolved in RNase-free water. RNase inhibitor was added to the dissolved solution to a final concentration of 20 U/μl for storage.

### Tested Samples Incubated With Probes

**Figure 2:**
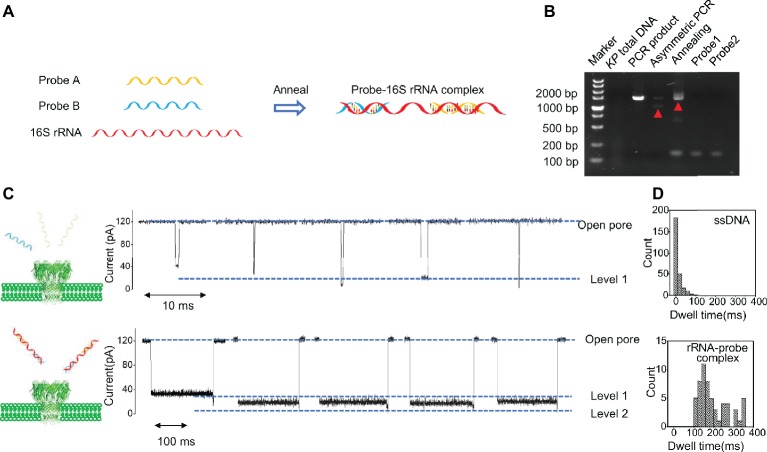
Probe-target RNA complex and its nanopore signal. **(A)** Probe A and probe B bound with 16S rRNA of carbapenem resistant KP. **(B)** Agarose gels result of probe A and probe B bound with CRKP 16S rRNA. **(C)** Signal of single-strand nucleic acid translocation and probe-16S rRNA complex translocation. **(D)** Dwell time distribution of the translocation events.

Probe A and probe B were synthesized by Sangon Biotech (Probe A: AGCAC AG AGA GCTTG CTCTC GGGTG ACGAG CGGCG GACGG GTGAG TAATG TCTGG GAAAC TGCCT GATGG AGGGG GATAA CTACT GGAAA CGGTA GCTAA TACCG CATAA CGTCG CAAGA CCAAG T, probe B: CCTTG AGGCG TGGCT TCCGG AGCTA ACGCG TTAAA TCGAC CGCCT GGGGA GTACG GCCGC AAGGT TAAAA CTCAA ATGAA TTGAC GGGGG CCCGC ACAAG CGGTG GAGGA TGTGG TTTAA TTCGA TGC). The probe A and probe B were annealed with tested samples using a PCR program (cooling of temperature from 95 to 4°C in 60 min) (Figure 2A). We used the agarose gel electrophoresis to verify the formation of probe A-probe B-target 16S rRNA complex.

### Nanopore Electrophysiological Experiment

The nanopore detection experiment was carried out in the chamber provided by Warner Instrument. The nanopore electrophysiological assay experiments were carried out under 150 mV bias voltage. The conductive buffer solutions at both -*cis* side and -*trans* side are 400 mM KCl with 10 mM HEPES, pH 7.0. The bilayer lipid membrane (BLM) that painted on both sides of the 150 μm hole was formed by 1,2-diphytanoyl-sn-glycero-3-phosphocholine (DPHPC) (Montal and Muellertj, 1972). MspA was added to the solution in the -*cis* chamber, allowing MspA proteins to insert after BLM formation. A single MspA nanopore insertion will cause a current increase corresponding to a conductance of *ca.* 1.2 nS. Samples were added into the -*cis* side after the insertion of a single MspA nanopore current signals were recorded by Heka EPC-10 patch clamp (HEKA).

### Data Analysis

We performed data analysis using software Clampfit 10.6 and Origin Pro 8.0. The current blockage was defined as Δ*I*/*I*_0,_ where *I*_0_ was the current of a fully opened pore, and Δ*I* was the amplitude of current blockage caused by translocated molecules. The dwell time was collected by the single-channel search feature of the Clampfit 10.6. Those two parameters were applied for quantitative analysis of target 16S rRNA from the surviving live *K. pneumoniae* resistant to carbapenem. All the data were obtained from a 20-min electrophysiological recording, and the experimental group was repeated three times independently.

### Clinical Specimens

Blood samples from 20 patients with KP infection were provided by the Department of Laboratory Medicine, West China Hospital of Sichuan University. This study was carried out in accordance with the recommendations of China National Measures for the Ethical Review of Biomedical Research Involving Humans and WMA Declaration of Helsinki. The protocol was approved by the Biomedical Ethics Committee of West China Hospital, Sichuan University.

The research used leftover specimens, that is, remnants of specimens collected for routine clinical care or analysis that would have been discarded, and complied with the criteria for a waiver of informed consent. The waiver for informed consent was granted by Biomedical Ethics Committee of West China Hospital, Sichuan University.

## Results

### Detection of 16S rRNA-Probe Complex

The result of agarose gel electrophoresis indicated that the rRNA-Probe complex was obtained successfully (Figure 2B). The dwell time of 16S rRNA-probe complex translocation signal in the carbapenem-resistant KP sample was in the range of 100–400 ms (Figures 2C,D) with a peak value of 196.98 ms, and the dwell time of single-stranded DNA translocation signal was in the range of 0–100 ms (Figures 2C,D) with a peak value of 12.03 ms. The dwell time of probe A and probe B was in the range of 0–70 ms (Supplementary Figure S1). Those results indicated that the signal with long dwell-time was attributed to the 16S rRNA-probe complex.

### Optimization of Bacterial Concentration and Standard Sample Test

**Figure 3:**
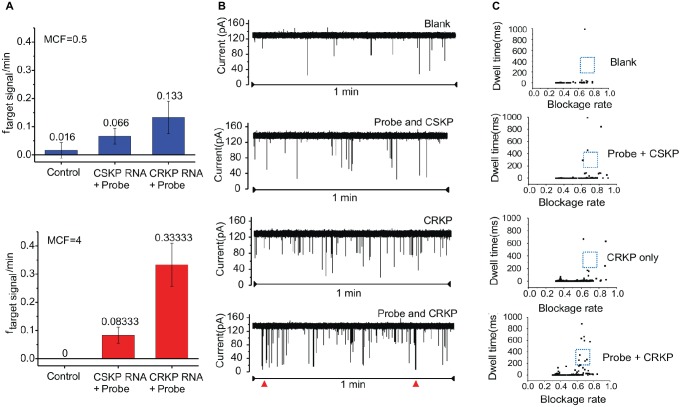
Distinguishing CRKP from CSKP from single channel recording signals. **(A)** Nanopore assay efficiency of 0.5 MCF sample and 4 MCF sample cultured for 4 h (*n* = 3 for each measurement). **(B)** Nanopore signal of control group, probe with carbapenem-sensitive KP, carbapenem-resistant KP, probe with carbapenem-resistant KP, respectively. **(C)** Scatter plot of translocation signal of different samples.

Two concentrations of KP were used to optimize the detection efficiency. In the samples of 0.5 MCF, the target RNA translocation signal frequency of the control group was 0.02 ± 0.02 per minute (*n* = 3), and the target RNA translocation signal frequency of CRKP group is 0.13 ± 0.05 per minute (*n* = 3) (Figure 3A). While in the samples of 4 MCF, target RNA translocation signal frequency of control group is 0 per minute (*n* = 3), and the translocation frequency of CRKP group is 0.33 ± 0.07 per minute (*n* = 3) (Figure 3A). Compared with 0.5 MCF sample, the 4 MCF sample showed improved detection performance in the nanopore assay.

The total RNA from carbapenem-resistant KP and carbapenem-sensitive KP were incubated with probe A and probe B, and the incubation solution was detected by MspA nanopore, respectively (Figure 3B). The two parameters of the obtained signals, blockage rate, and dwell time, from nanopore assay of the samples were plotted into scattering plot (Figure 3C), and obvious difference in dwell time between different group can be observed, especially in the range of blockage rate 0.6–0.8 and dwell time 100–400 ms. Thus, signals in this range were chosen as specific signals for diagnosis. After comparing the number of 16S rRNA-probe signals in the given range from blank, control, CSKP and CRKP samples, *f* = 0.1 min^−1^ was used as the target events threshold to distinguish the carbapenem resistance of *K. pneumoniae*. In order to determine the minimum bacteria culture time needed to distinguish CSKP and CRKP, samples with different bacteria culture time including 2, 4, and 8 h were tested by MspA nanopore. Those results indicated that 4 h is the optimal bacteria culture time in considering both sensitivity and efficiency.

### Double-Blind Test of Clinical Samples by MspA Nanopore

Based on the above results of standard CRKP and CSKP samples, further experiments were performed to verify the potential application of this method in clinical diagnosis. Bacteria in the blood samples from 20 patients with KP infection provided by the West China Hospital were cultured and the total RNA were extracted and used for double-blind experiments. Each sample was detected by MspA nanopore at least three times. After analysis, the number of 16S rRNA-probe signals within the blockage rate of 0.6–0.8 and dwell time of 100–400 ms were collected and compared with target events frequency threshold *f*_threshold_.

**Figure 4:**
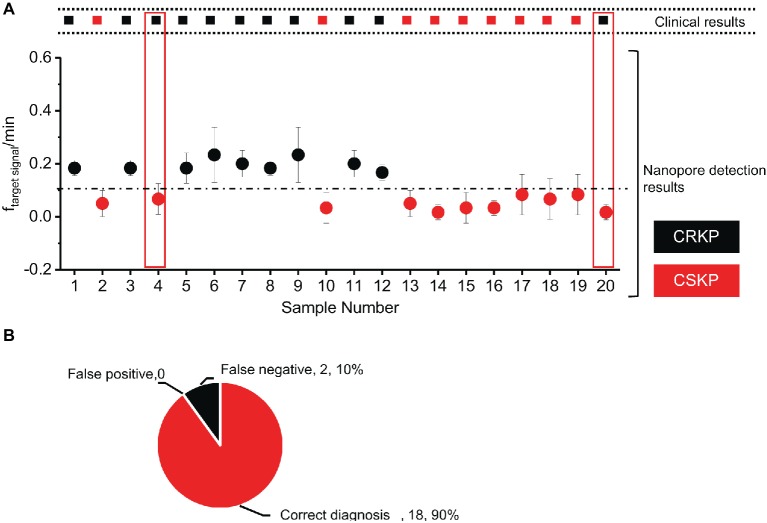
Double-blind test of clinical samples and evaluation of the assay accuracy. **(A)** Nanopore assay of 20 *Klebsiella pneumoniae* clinical samples. **(B)** Accuracy rate of the nanopore assay method. (*n* = 3 for each measurement).

Out of the 20 samples, nine of them were above the threshold (0.1·min^−1^) and were identified as carbapenem-resistant KP. The other 11 samples showed a lower target event frequency below 0.1·min^−1^, and these clinical samples were identified as carbapenem-sensitive KP (Figure 4A). Compared with the assay results obtained from standard clinical (disk diffusion method or PCR) method (Supplementary Tables S1, S2), results of 18 samples by nanopore assay were correct (Figure 4B), while two showed false negative results.

## Discussion

Accurate and fast diagnosis of KP’s carbapenem resistance is quite important in the clinical therapy process. Our objective is to develop a new KP carbapenem resistant detection method based on nanopore sensing technology to provide a potential solution to the challenges of the present methods that cannot fully meet the clinical demands.

Protein nanopore expression, purification and electrophysiological assay technology has been fast developed during the past 20 years, and various types of protein nanopores have been constructed for well-established nucleic acids assay (Haque et al., 2013). In this experiment, we designed two DNA probes to specifically bind to the 16S rRNA of carbapenem-resistant KP; the 16S rRNA-probe complex translocation through MspA nanopore will cause a much slower translocation dwell time between 100 and 400 ms. Based on the dwell time and blockage of translocation signals, we can figure out targeted 16S rRNA of carbapenem-resistant KP samples (Supplementary Figure S2). Both carbapenem resistant KP standard samples and carbapenem sensitive KP standard samples were detected, and the result showed that this method can be used to distinguish the carbapenem resistant KP and carbapenem sensitive KP with only 4 h of bacteria culture. Furthermore, 20 clinical samples provided by West China Hospital were assayed by MspA nanopore. Among the 11 carbapenem resistant KP clinical samples, nine samples were diagnosed correctly, while two samples were detected as false negative; among the nine carbapenem sensitive KP samples, nine samples were diagnosed correctly. The accuracy rate of nanopore diagnose method is 90%. RNA degradation during sample storage or transfer was the main reason leading to the 10% false negative diagnosis. Variation of the transportation of the clinical samples from hospital to research lab and time gap between the sample processing and nanopore assay increased the possibility of RNA degradation, leading to the decrease number of 16S rRNA and specific signals detected by nanopore.

The above results was a pilot and experimental study of new single-molecule diagnostic technology, and further validation studies aiming to increase the sensitivity could be carried out in several aspects: optimization of bacterial culture condition and RNA extraction; modification and reengineering of the protein nanopore for the 16S rRNA-probe complex detection; test with larger number of clinical samples; and improved statistics methods for data processing. Stable storage of the aliquoted MspA protein at −80°C enabled the batch production of the nanopore for future multiple tests.

In conclusion, our research provided a proof of concept nanopore sensing technology with potential application in the rapid clinical diagnosis of carbapenem resistant KP and carbapenem sensitive KP. Compared with the disk diffusion method or PCR method, two of the most widely used methods in the clinical diagnosis, nanopore sensing is a low-cost, efficient, and easy-operational method (Table 1). This method could be a complement to the existing diagnosis methods, thereby contributing to the clinical laboratory diagnosis. With the development of lab-on-chip nanopore devices (Quick et al., 2016), rapid detection of multiple clinical samples using nanopore array in an integrated POCT device in hospital could be realized.

**Table 1:** Comparison of different CRKP detection methods.

Method	Accuracy	Sample preparation	Analysis time	Costs	Reference
Phenotypic analysis	Middle	18–24 h	5 min	Low	van Dijk et al., 2014; Kim et al., 2016
PCR	Middle	3–4 h	<4 h	Middle	Schechner et al., 2009; Hindiyeh et al., 2011
Real-time PCR	High	3–4 h	4–6 h	High	Nijhuis et al., 2013; Frickmann et al., 2014
TaqMan PCR	High	3–4 h	<2 h	High	Swayne et al., 2013
Nanopore assay	Middle	6.5 h	1 h	Low	This study

## Data Availability

All datasets generated for this study are included in the manuscript and the Supplementary Files.

## Ethics Statement

Blood samples from 20 patients with KP infection were provided by the Department of Laboratory Medicine, West China Hospital of Sichuan University. This study was carried out in accordance with the recommendations of China National Measures for the Ethical Review of Biomedical Research Involving Humans and WMA Declaration of Helsinki. The protocol was approved by the Biomedical Ethics Committee of West China Hospital, Sichuan University. The research used leftover specimens, that is, remnants of specimens collected for routine clinical care or analysis that would have been discarded, and complied with the criteria for a waiver of informed consent. The waiver for informed consent was granted by Biomedical Ethics Committee of West China Hospital, Sichuan University.

## Author Contributions

JG and MK designed the research. HN, CZ, and PZ took part in the experiment design. WZ, QL, YL, and SW performed total RNA extraction. HN, LW, ML, and HL finished the nanopore assay experiment. JG and MK wrote the manuscript. HN, CZ, LW, and QY assisted the manuscript preparation.

### Conflict of Interest Statement

The authors declare that the research was conducted in the absence of any commercial or financial relationships that could be construed as a potential conflict of interest.
