# Supplementary material for: Rapid Nanopore Assay for Carbapenem-Resistant Klebsiella pneumoniae
Source: Front Microbiol. 2019 Jul 30;10:1672. doi: 10.3389/fmicb.2019.01672 (PMC6682601; doi:10.3389/fmicb.2019.01672)
Supplement: Supplementary file 1 [file Data_Sheet_1.pdf]

Supplementary Materials

Rapid Nanopore Assay for Carbapenem-resistant *Klebsiella Pneumoniae*

Haofu Niu[1,2†], Weili Zhang[1†], Liangwan Wei[2†], Meng Liu[2], Hao Liu[2], Changjian Zhao[1], Peng Zhang[1], Quanfeng Liao[1], Ya Liu[1], Qingyue Yuan[1], Siying Wu[1], Mei Kang[1 *], Jia Geng[1 *]

[1] *Department of Laboratory Medicine, State Key Laboratory of Biotherapy and Cancer Center, West China Hospital, Sichuan University and Collaborative Innovation Center, Chengdu 610041 China*

[2] *Department of Microbiology, West China School of Basic Medical Sciences & Forensic Medicine, Sichuan University, Chengdu 610041 China*

**Correspondence:*

Mei Kang

kangmei@sina.com

Jia Geng

geng.jia@scu.edu.cn

[†]These authors have contributed equally to this work.

Expression and purification of MspA nanopore

BL21 Competent *E. coli* is a widely used strain and is suitable for transformation and protein expression. The gene of MspA nanopore was cloned into the pET-28b plasmid, and the pET-28b plasmid carried with MspA gene was transferred into engineering bacteria *E.coli* BL21(Niederweis et al., 1999). Successfully transferred *E.coli* was cultured in the LB media at 37℃, and the kanamycin was added to 50 μg/mL. While the optical density (600 nm) was close to 0.8, the 0.8 mM IPTG was added into the LB (lysogeny broth) media with an inducing temperature of 15℃. After 12h of induction, the *E. coli* was collected through centrifugation. The supernatant was collected after the *E. coli* was broken by the ultrasonicator, and further purified by anion-exchange column (Q-Sepharose) and molecular sieve (Superdex 200 16/90). The purified protein was analyzed by 10% SDS-PAGE (Sodium dodecyl sulfate polyacrylamide gel electrophoresis).

The purified MspA nanopore protein can be aliquoted and stored at − 80 °C. The aliquots remain stable for years, and the nanopore remains active upon thaw (Haque et al., 2013).

Probe design

We design probes to combine with specific fragments of 16S rRNA, so that we can recognize specific signals regarding the target nucleic acid through nanopore. Since the target 16S rRNA has a long length of 932 bp, it is difficult to distinguish the 16S rRNA-probe complex without probes or by a single probe, thus two probes were designed to bind the specifically expressed 16S rRNA of KP (**Figure 2A**). Probe A bound to the conserved area of 16S rRNA (810 nt-937 nt) was used to help the 16S rRNA-probe complex keep consistent secondary structure and increase dwell time which can be distinguished easily by MspA ; Probe B bound to the specific area of 16S rRNA (10 nt-136 nt) has been reported to be 100% specific and we used it to increase the specificity (Poh et al., 2004).

REFERENCES

Niederweis, M., Ehrt, S., Heinz, C., Klöcker, U., Karosi, S., Swiderek, K. M., et al. (1999). Cloning of the mspA gene encoding a porin from Mycobacterium smegmatis. *Mol. Microbiol.* 33, 933–945. doi:10.1046/j.1365-2958.1999.01472.x.

Poh, C. L., Kurupati, P., Chow, C., and Kumarasinghe, G. (2004). Rapid Detection of Klebsiella pneumoniae from Blood Culture Bottles by Real-Time PCR. *J. Clin. Microbiol.* 42, 8–12. doi:10.1128/JCM.42.3.1337.

Table S1. **Clinical sample information of CRKP.** The Sample ID is the patient ID in hospital and the Sample number is the corresponding number in our study.

Sample ID	Sample #	SCIM (mm)	Drug resistance gene	Drug resistance gene
**17012889-3**	1	6	KPC	KPC-2
**17019349-3**	3	6	KPC	KPC-2
**1810143046**	4	6	KPC	KPC-2
**15043287-1**	5	6	KPC	KPC-2
**15057156-1**	6	6	KPC	KPC-2
**15083593-1**	7	6	KPC	KPC-2
**1807191036**	8	6	KPC	KPC-2
**1807271015**	9	24	Negative	-
**17008404-1**	11	6	KPC	None
**17012837-3**	12	6	KPC	KPC-2
**17020362-3**	20	6	KPC	KPC-2

Table S2. Clinical sample information of CSKP. The Sample ID is the patient ID in hospital and the Sample number is the corresponding number in our study.

Sample ID	Sample #	Detected bacteria	Drug-resistant form	SCIM-IPM (mm)
**1809163029**	2	CSKP	ESBL	34
**1807171076**	10	CSKP	ESBL	33
**1807163074**	13	CSKP	-	-
**1806163019**	14	CSKP	-	-
**1807043013**	15	CSKP	ESBL	33
**1806301100**	16	CSKP	ESBL	34
**1806133062**	17	CSKP	-	-
**1806083069**	18	CSKP	ESBL	32
**1806163021**	19	CSKP	ESBL	31

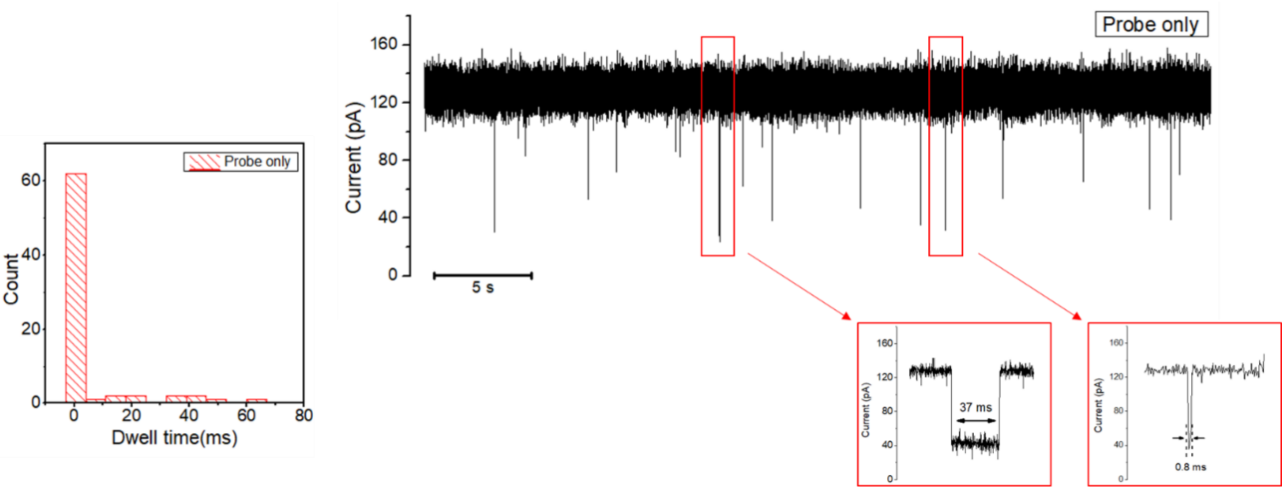

Figure S1. **Translocation signal of probe only group**. The dwell time of the DNA probe translocation through nanopore is in the range of 0 to 70 ms.

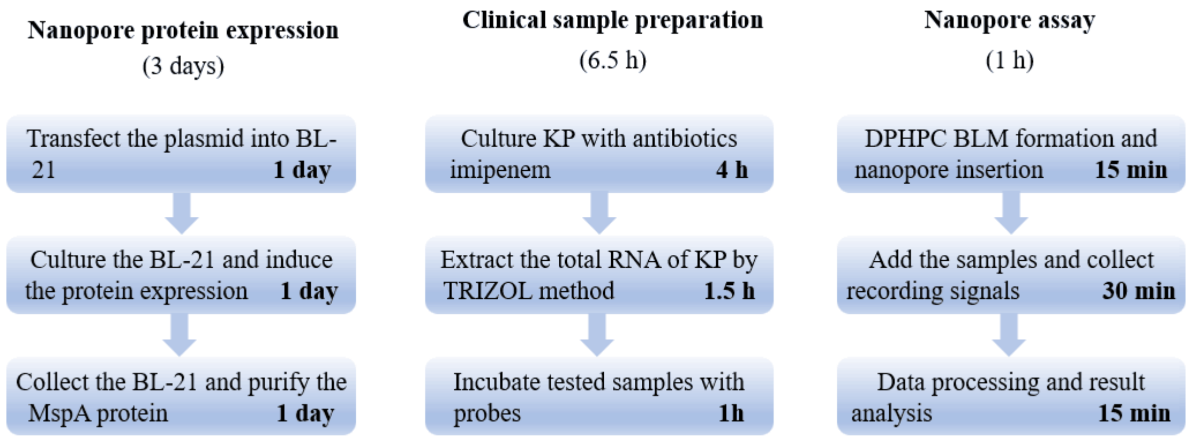

Figure S2. **Flow chart of the experiment and time cost in total.** The MspA protein is stored in the refrigerator at -80°C for use after expression and purification for further test. There is no need to prepare the nanopore for each experiment.
